# Effects of Roasting Temperatures on Peanut Oil and Protein Yield Extracted via Aqueous Enzymatic Extraction and Stability of the Oil Body Emulsion

**DOI:** 10.3390/foods12224183

**Published:** 2023-11-20

**Authors:** Yajing Zhang, Yu Chen, Chen Liu, Fusheng Chen, Lijun Yin

**Affiliations:** 1 College of Food Science and Engineering, Henan University of Technology, Zhengzhou 450001, China; zyj220521@163.com (Y.Z.); loyyer@163.com (Y.C.); liuchen919258@163.com (C.L.); ljyin@cau.edu.cn (L.Y.); 2 Food Laboratory of Zhongyuan, Luohe 462300, China; 3 College of Food Science and Nutritional Engineering, China Agricultural University, Beijing 100083, China

**Keywords:** peanut, roast, aqueous enzymatic extraction, emulsion, stability, oil quality

## Abstract

Oil body emulsions (OBEs) affect the final oil yield as an intermediate in the concurrent peanut oil and protein extraction process using an aqueous enzyme extraction (AEE) method. Roasting temperature promotes peanut cell structure breakdown, affecting OBE composition and stability and improving peanut oil and protein extraction rates. Therefore, this study aimed to investigate the effects of pretreatment at different roasting temperatures on peanut oil and protein yield extracted through AEE. The results showed that peanut oil and protein extraction rates peaked at 90 °C, 92.21%, and 77.02%, respectively. The roasting temperature did not change OBE composition but affected its stability. The OBE average particle size increased significantly with increasing temperature, while at 90 °C, the zeta potential peaked, and the interfacial protein concentration hit its lowest, indicating OBE stability was the lowest. Optical microscopy and confocal laser scanning microscopy confirmed the average particle size findings. The oil quality obtained after roasting treatment at 90 °C did not differ significantly from that at 50 °C. The protein composition remained unaffected by the roasting temperature. Conclusively, the 90 °C roasting treatment effectively improved the yield of peanut oil extracted using AEE, providing a theoretical basis for choosing a suitable pretreatment roasting temperature.

## 1. Introduction

Peanuts rank as a major oilseed crop globally, with abundant oil and protein content. According to a United States Department of Agriculture report, the projected global consumption of peanut oil will reach 6.283 million metric tons between 2022 and 2023 [1]. Peanut oil is rich in various unsaturated fatty acids, while peanut protein is abundant in eight essential amino acids vital for the human body. These attributes make them a widely sought-after plant protein resource in the food industry [2]. Presently, the prevailing techniques for peanut oil processing, both domestically and internationally, involve pressing and solvent extraction. However, the high energy consumption of the pressing method and extrusion of raw materials compromise the nutritional and functional properties of proteins, resulting in significant protein resource wastage. While the solvent extraction method yields a high extraction rate, it comes with the challenge of solvent residues in the oil and grease, necessitating rigorous refining procedures. Additionally, the process exposes the human body to a substantial volume of organic reagents, leading to mild reactions in the central nervous system, including dizziness, nausea, and headaches, which are hazardous to human safety [3]. Aqueous enzyme extraction (AEE) employs water as a medium, leveraging both mechanical shear force and biological enzymes to break down the peanut cell wall and its internal structure. This approach enables the synchronous separation of oil and protein [4]. Compared to the pressing and solvent extraction methods, AEE, as a novel green processing technique, offers distinct advantages such as product safety, low energy consumption, and environmental protection. This method can simultaneously produce healthy oils and proteins, rendering it suitable for use in commercial oil processing [5]. Furthermore, while AEE holds considerable potential, it currently falls short based on cell wall destruction and cell dissociation efficiency, thereby affecting oil yield and hampering its industrial development. Roasting pretreatment is conducive to the rupture of the cell wall structure of the oilseed, increasing the surface contact area between the oilseed and the enzyme, enhancing the dissolution of intracellular substances, and promoting small oil droplet aggregation, collectively contributing to improved oil yield [6,7]. Additionally, roasting facilitates the removal of the red coat from peanuts, thus improving the quality of oil and protein while reducing refining costs [8].

Peanut oils and proteins exist as oil bodies (OBs) and protein bodies [9]. OBs, also known as greasy bodies, lipid droplets, and spheres, serve as storage lipids in oilseeds, legumes, and nuts [10]. These OBs take on a spherical shape with a core of neutral lipids encircled by a half-unit membrane composed of phospholipids and endogenous proteins [9,10]. It was found that the ratio of oil body interface proteins (OBIPs) to phospholipids affects the interfacial structure, which in turn affects the nature and stability of the reconstituted OB, suggesting that the structure and composition of the OB is a key factor affecting its stability [11,12]. OBIPs are categorized into endogenous and exogenous proteins, with the former comprising oleosin, caleosin, and steroleosin. Oleosins represent a class of hydrophobic, alkaline, small-molecule proteins with a molecular weight ranging from 15 to 26 kDa. They possess dual functionality, serving not only as monoglyceridyltransferases but also playing an important role in phospholipase activity. The molecular weight range of Caleosin is 27–35 kDa, which not only stabilizes OB but also acts as a peroxygenase, catalyzing the production of oxidized polyunsaturated fatty acids. Steroleosin, also known as sterol dehydrogenase, dehydrogenates plant sterols into ketone derivatives with a molecular weight of 40–55 kDa [13,14]. During the AEE of peanut oil and protein, exogenous proteins adhere to the OB surface, forming a multilayered membrane held together by various forces. This process affects the stability of the OB and directly affects its performance in the subsequent emulsion-breaking studies [15,16]. Zhao et al. [15] found substantial quantities of exogenous proteins in crude OBs from sunflower, sesame, walnut, jicama, and rapeseed. Furthermore, they discovered that, under specific conditions, unknown endogenous proteases hydrolyze oleosin and exogenous proteins. In addition, Liu et al. [16] observed that exogenous proteins, such as lipoxygenase, arachin, conarachin, and ferritin, adhere on the surface of peanut OBs through weak interactions, including electrostatic forces, hydrophobicity, and hydrogen bonding. These interactions affect the stability of the oil bodies. The phospholipids constituting the OB membrane can be categorized into major and minor phospholipids. Major phospholipids encompass phosphatidylcholine and phosphatidylserine, while minor phospholipids comprise phosphatidylinositol, phosphatidylethanolamine, and phosphatidyl acid [17]. Variations influence the stability of the interfacial membrane in the composition and content of phospholipids. These differences arise from the choice of plant species and extraction methods.

In the process of simultaneous extraction of peanut oil and protein through AEE, it is beneficial for oil body emulsions (OBEs) to exhibit a lower stability because it enhances the release of peanut oil. To boost the yield of concurrently extracted peanut oil and protein using AEE, increasing the degree of peanut cell wall disruption is crucial. This enhancement facilitates the release of oil and protein while reducing the stability of OBEs. Therefore, this study aims to analyze peanuts subjected to different roasting temperatures. This analysis will facilitate the comparison of the effects of roasting temperature on the distribution of peanut oil and protein across various phases. Additionally, the changes in the composition and stability of OBEs were evaluated, as was the quality of peanut oil and protein composition. This study provides a theoretical basis for selecting the appropriate pre-roasting temperature.

## 2. Materials and Methods

### 2.1. Materials

Peanuts (Yuhua 23) were purchased from the Henan Academy of Agricultural Sciences (Zhengzhou, China). Pressed oil was produced using hydraulic equipment at 45 Mpa for 30 min (YKY-6YL-550, Bafang Machinery Equipment Co., Ltd., Zhengzhou, China). Leaching oil (n-hexane extraction) was extracted using the Soxhlet extraction method at 60 °C for 8 h. Commercial oil purchased from local market (Zhengzhou, China). Viscozyme^®^ L (5086 U/mL, a multi-enzyme complex, including arabinase, cellulase, β-Dextranase, hemicellulase, and xylanase) was procured from Novozymes Co., Ltd. (Shanghai, China). All other reagents and chemicals were sourced from Sigma-Aldrich Trading Co., Ltd. (Shanghai, China).

### 2.2. Aqueous Enzymatic Extraction of Peanut Oil and Protein

**Figure 1:**
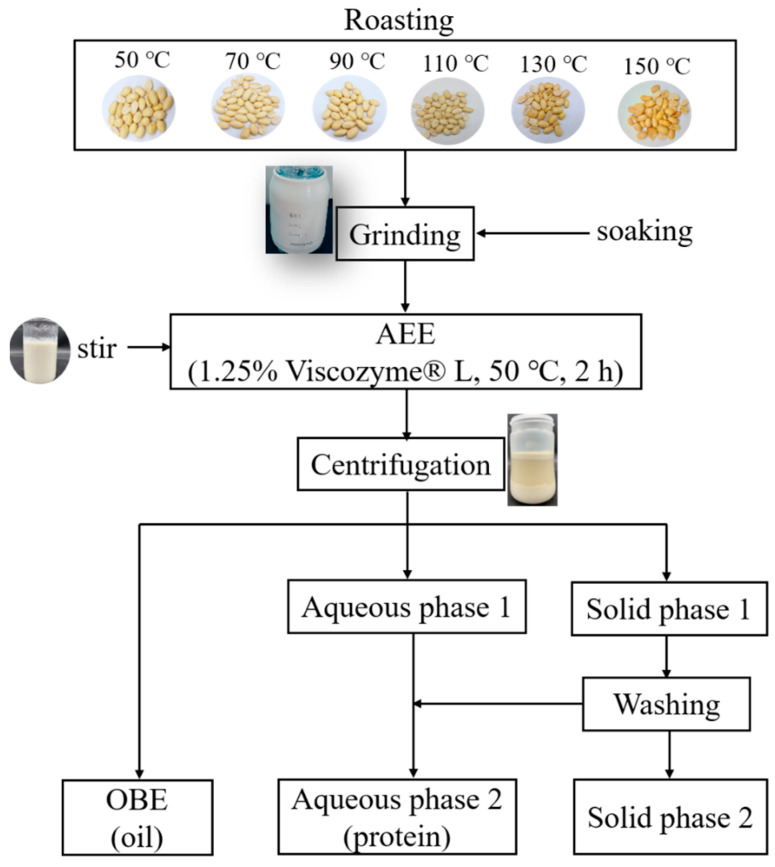
Flow chart of AEE for peanut oil and protein.

Peanut oil and protein were prepared using the methods described by Liu [18] and Zhao et al. [19] with minor modifications. Figure 1 presents a flowchart of the AEE for peanut oil and protein. For the de-red-coating treatment, the peanuts were roasted in an electric thermostatic drying oven (DHG-9246A, Jinghong Co., Ltd., Shanghai, China) at 50 °C, 70 °C, 90 °C, 110 °C, 130 °C, and 150 °C for 30 min. Peanuts (40 g) subjected to varying roasting temperatures were soaked in deionized water (*w*/*v*, 1:4) and stored at 4 °C for 8 h. Subsequently, the mixture was ground for 120 s using a multifunctional grinder (C022E, Joyoung Co., Ltd., Shandong, China). Following this, Viscozyme^®^ L was introduced, and the mixture was stirred for 2 h at 50 °C to ensure complete enzymatic processing. The solution was cooled and then centrifuged at 4000× *g* for 15 min (DZ267-32C6, Anting Scientific Instrument Factory, Shanghai, China), resulting in the separation of the upper OBE, intermediate aqueous phase 1, and lower solid phase 1. To wash solid phase 1, the volume of deionized water was added thrice, and the resulting washed supernatant was combined with aqueous phase 1, yielding aqueous phase 2. The lower layer constituted solid phase 2. Freeze-dried aqueous phase 2 was used to obtain peanut protein through AEE.

### 2.3. Analysis of the Main Composition of OBE

The moisture, protein, and phospholipid levels in the OBE were assessed using the drying method, Kjeldahl method (conversion factor, 5.46), and molybdenum blue colorimetry, respectively [16,18]. The oil content in the OBEs was determined using the chloroform–methanol method described by Liu et al. [4] with appropriate modifications as necessary. The OBE was mixed with a chloroform–methanol solution (2:1, *v*/*v*) at a ratio of 1:3 (*w*/*v*) and stirred for 3 h. After centrifugation, the extraction solution and filtrate were retrieved, and an additional chloroform–methanol solution was added to the filtrate and stirred for an additional 3 h. The extraction process was repeated thrice, and the resulting extracts were combined. The solvent was eliminated from the extraction solution through rotary evaporation (60 °C, vacuum degree > 0.06 MPa), and it was subsequently dried in a 50 °C electric thermostatic drying oven for 10 h to obtain total fat. The oil content of the solid phase was determined using the Soxhlet extraction method (n-hexane extraction) after freeze drying, while the oil content in the aqueous phase was determined using the alkaline hydrolysis method [18]. The extraction rates of peanut oil and protein were calculated following Equations (1) and (2). The residual oil rate in the aqueous phase was calculated using Equation (3), and the residual oil and protein rates in the solid phase were calculated using Equations (4) and (5).

(1) $$\mathrm{Oil}\;\mathrm{yield}\left(\%\right)=\frac{\mathrm{OBE}\;\mathrm{weight}\left(g\right)\times \mathrm{oil}\;\mathrm{content}\;\mathrm{of}\;\mathrm{OBE}\left(\%\right)}{\mathrm{weight}\;\mathrm{of}\;\mathrm{peanuts}\left(g\right)\times \mathrm{oil}\;\mathrm{content}\;\mathrm{of}\;\mathrm{peanuts}\left(\%\right)}\times 100\;$$

(2) $$\mathrm{PPER}\left(\%\right)=\frac{\mathrm{APW}\left(g\right)\times \mathrm{protein}\;\mathrm{content}\;\mathrm{in}\;\mathrm{aqueous}\;\mathrm{phase}\left(\%\right)}{\mathrm{weight}\;\mathrm{of}\;\mathrm{peanuts}\left(g\right)\times \mathrm{protein}\;\mathrm{content}\;\mathrm{of}\;\mathrm{peanuts}\left(\%\right)}\times 100$$

(3) $$\mathrm{APROR}\left(\%\right)=\frac{\mathrm{APW}\left(g\right)\times \mathrm{oil}\;\mathrm{content}\;\mathrm{of}\;\mathrm{aqueous}\;\mathrm{phase}\left(\%\right)}{\mathrm{weight}\;\mathrm{of}\;\mathrm{peanuts}\left(g\right)\times \mathrm{oil}\;\mathrm{content}\;\mathrm{of}\;\mathrm{peanuts}\left(\%\right)}\times 100$$

(4) $$\mathrm{SPROR}\left(\%\right)=\frac{\mathrm{solid}\;\mathrm{phase}\;\mathrm{weight}\left(g\right)\times \;\mathrm{oil}\;\mathrm{content}\;\mathrm{of}\;\mathrm{solid}\;\mathrm{phase}\left(\%\right)}{\mathrm{weight}\;\mathrm{of}\;\mathrm{peanuts}\left(g\right)\times \mathrm{oil}\;\mathrm{content}\;\mathrm{of}\;\mathrm{peanuts}\left(\%\right)}\times 100$$

(5) $$\mathrm{SPRPR}\left(\%\right)=\frac{\mathrm{solid}\;\mathrm{phase}\;\mathrm{weight}\left(g\right)\times \;\mathrm{protein}\;\mathrm{content}\;\mathrm{of}\;\mathrm{solid}\;\mathrm{phase}\left(\%\right)}{\mathrm{weight}\;\mathrm{of}\;\mathrm{peanuts}\left(g\right)\times \mathrm{protein}\;\mathrm{content}\;\mathrm{of}\;\mathrm{peanuts}\left(\%\right)}\times 100$$

where PPER (%) is the peanut protein extraction rate, APW (g) denotes the aqueous phase weight, APROR (%) represents the aqueous phase residual oil rate, SPROR (%) is the solid phase residual oil rate, and SPRPR (%) is the solid phase residual protein rate.

### 2.4. Physical Properties

#### 2.4.1. Determination of Particle Size Distribution and Zeta Potential

The OBE particle size distribution and zeta potential were examined following the procedures outlined by Xu et al. [20] with minor adjustments. The particle size was expressed as De Brouckere volume-weighted average diameter (D_4,3_) and Sauter surface-weighted average diameter (D_3,2_). The OBE was diluted in deionized water at a ratio of 1:100 (*w*/*v*), vortexed and shaken for 2 min, and then analyzed using a laser particle size analyzer (BT-9300H, Dandong Baxter Instrument Co., Ltd., Dandong, China) in automatic measurement mode.

The refractive index of the sample was set to 1.46, while that of the medium was 1.33. Particle size distribution measurements for the OBE were conducted automatically when the laser obscuration of the sample fell within the range of 5% and 10%; the instrument has a cycle speed of 1600 r/min. To determine the zeta potential of the OBEs, 1 g of OBE was diluted by a factor of 1000, utilizing a phosphate buffer (10 mmol/L, pH 7.0). The solution was then vortexed, shaken for 2 min, and equilibrated for 120 s using a zeta potential analyzer (Zeta sizer Nano ZSP, Marvin Instrument Co., Ltd., Marvin, UK).

#### 2.4.2. Measurement of Surface Protein Concentration

Surface protein concentration (Γ) was determined using the method reported by Chabrand et al. [21] with some modifications. It was subsequently calculated using Equation (6).

(6) $$\Gamma =\frac{M_{p/o}}{\mathrm{SSA}},\;\mathrm{SSA}=\frac{6}{D_{3,2}\times \rho_{\mathrm{oil}}}$$

Γ (mg/m^2^) denotes the surface protein concentration; M_p/o_ represents the surface-protein-to-fat-mass ratio of the OBE (mg/g); SSA is the surface area of the OBE (m^2^/g); D_3,2_ signifies the Sauter surface-weighted average diameter of the OBE (10^−6^ m); and ρ_oil_ denotes that peanut oil has a density of 0.91 × 10^6^ g/m^3^. 

### 2.5. Microstructure Observation

#### 2.5.1. Optical Microscopy

The OBEs extracted from peanuts roasted at various temperatures were diluted in deionized water at a ratio of 1:50 (*w*/*v*), thoroughly mixed, and then 10 μL was pipetted onto slides using a pipette gun. A coverslip was placed over the droplet to prevent air bubbles, and the sizes and distributions of the droplets were observed using an optical microscope (CX33, Olympus, Tokyo, Japan).

#### 2.5.2. Confocal Laser Scanning Microscopy (CLSM)

Fluorescent dyes and staining methods were employed, as previously described by Li et al. [22], with slight modifications. To stain the OBEs, they were initially diluted 50-fold using deionized water. Subsequently, 1 mL of the diluted OBE was combined with 10 μL of Nile red and thoroughly mixed. Following this, 10 μL of the stained samples were pipetted into fluted slides, ensuring the use of coverslips to prevent the generation of air bubbles and solution overflow, as observed using CLSM (FV3000, Olympus, Tokyo, Japan).

#### 2.5.3. Transmission Electron Microscopy (TEM)

The peanut microstructure was examined based on the method described by Cao et al. [23] with some modifications. Peanuts at different roasting temperatures were cut into 1–2 mm pellets and immersed in a 2.5% (*v*/*v*) glutaraldehyde sodium phosphate buffer (0.1 mol/L, pH 7.2) at 4 °C overnight. The fixative was removed, and the samples were rinsed thrice for 15 min each with sodium phosphate buffer (0.1 mol/L, pH 7.2). Following this, the samples were fixed with 1% (*w*/*v*) osmium acid solution for 1–2 h and then rinsed with sodium phosphate buffer. Subsequently, the samples were dehydrated with graded concentrations of ethanol, each lasting 15 min, followed by a 20 min treatment with 100% ethanol. Finally, they were dehydrated for 20 min with pure acetone. Following dehydration, the samples were infiltrated and embedded in Spur resin, and subsequently, 70–90 nm thick slices were obtained using an ultrathin slicing machine. The slices were stained with lead citrate and acetic acid dioxygen axis for 5–10 min. Following staining, they were dried before being observed, and the images were recorded using a TEM (HT7800, Hitachi, Tokyo, Japan) with an accelerated voltage of 90 kV.

### 2.6. Evaluation of Oil Quality

#### 2.6.1. Measurement of Basic Indicators

Extraction of aqueous enzyme peanut oil from peanut OBEs was achieved using the chloroform–methanol method described in Section 2.3. The acid value and peroxide value of peanut oil were assessed using the AOCS official methods Cd 3d-63 and Cd 8-53 [24]. To determine the oxidation stability, the oxidation induction time was measured using a Rancimat instrument (Metrohm CH series743, Zofingen, Switzerland). This involved continuous observation of the changes in pure water conductivity of peanut oil under a heating temperature of 120 ± 0.2 °C and an airflow rate of 20 L/h [25].

#### 2.6.2. Determination of Tocopherol Content

A total of 0.5 g of the oil sample was accurately measured and placed in a 10 mL volumetric flask. Chromatographically pure n-hexane was added to adjust the volume to the scale, thoroughly mixed. The sample was subsequently filtered through a 0.45 μm organic membrane into a liquid-phase vial. The mobile phase employed was hexane/isopropanol (99:1, *v*/*v*) with a flow rate of 0.8 mL/min. The High-Performance Liquid Chromatography (1260 Infinity II, Agilent Technologies Inc., Santa Clara, CA, USA) conditions were consistent with those described by Ji et al. [26].

#### 2.6.3. Determination of Fatty Acid Composition

The oil samples were methyl-esterified. Firstly, 3 drops of oil and 2–3 zeolites were placed in a 50 mL round-bottomed flask. Subsequently, 6 mL of sodium methanol solution was added to the round-bottomed flask, which was connected to a condensation reflux device. Subsequently, 3 mL of boron trifluoride ethyl ether solution and 6 mL of methanol solution were introduced from the upper end of the condenser tube after boiling the liquid in the flask for 5 min and boiling it for an additional 1 min. Following this, 5 mL of n-hexane of chromatography grade was added through the upper end of the condenser tube and boiled for 1 min. The flask was removed, and after cooling, saturated NaCl solution was added, thoroughly mixed, and then left to stratify. The supernatant was aspirated and dried with anhydrous sodium sulfate. It was then centrifuged at 2000× *g* for 3 min and injected into the liquid-phase vial through a 0.45 μm organic filter membrane. The fatty acid composition was then analyzed using a gas chromatograph (7890A, Agilent Technologies Inc., Santa Clara, CA, USA) with the gas chromatographic conditions as previously described by Liu et al. [4].

### 2.7. Sodium Dodecyl Sulfate–Polyacrylamide Gel Electrophoresis (SDS-PAGE)

OBIPs were prepared as previously described by Zhou et al. [27] with slight modifications. The OBE protein content was determined using the Kjeldahl method. Deionized water was added following a material/liquid ratio of 1:3 (*w*/*v*). Sodium dodecyl sulfate was introduced at a protein-to-sodium dodecyl sulfate mass ratio of 1:1.5 (*w*/*w*). The mixture was then vortexed for 3 min and transferred to a high-speed freezing and centrifugation machine (10,000× *g*, 20 min, 4 °C). An intermediate aqueous phase was obtained through centrifugation. The protein content of the intermediate aqueous phase and aqueous phase 2 was determined by mixing with a reducing sample buffer to achieve a final protein content of 3 mg/mL. This was performed using 5% concentrate and 12% separator gels, followed by fixation with a fixative for 30 min, staining for 2 h, and decolorization until the background was clear.

### 2.8. Determination of Amino Acid Composition

The amino acid composition of the aqueous phase proteins was analyzed using the method previously described by Liu et al. [28] with minor adjustments. The lyophilized sample was accurately weighed and placed into an anaerobic tube. To perform this, 10 mL of HCl (6 mol/L) and 2–3 drops of phenol were added. The tube lid was promptly tightened after being filled with nitrogen for 5 min and then subjected to hydrolysis in an oven at 110 °C for 22 h. The sample was removed, allowed to cool, filtered, and then diluted into a 100 mL volumetric flask. From the filtrate, 1 mL was evaporated in the test tube concentrator. Following this, 1 mL of the sample diluent was added, and the mixture was subjected to 2 min ultrasonication. Finally, the sample was analyzed using an amino acid analyzer (S-433D, Sykam (Beijing) Scientific Instrument Co., Ltd., Beijing, China).

### 2.9. Statistical Analysis

All experiments were conducted at least three times, and the data were expressed as mean ± SD. One-way variance analysis (NOVA) and Duncan’s multiple-range test were carried out using SPSS 26.0 software. Origin 2023 software was used to plot the images.

## 3. Results and Discussion

### 3.1. Effect of Roasting Temperatures on the Extraction Rate of Peanut Oil and Protein

**Figure 2:**
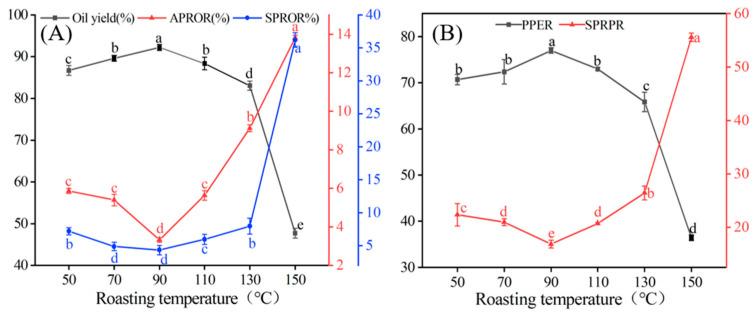
Distribution of oil and protein in different phases at different roasting temperatures. (**A**) the distribution of oil (**B**) and protein; APROP, the aqueous phase residual oil rate; SPROR, the solid phase residual oil rate; PPER, the peanut protein extraction rate; SPRPR, the solid phase residual protein rate. A significant difference between samples is indicated by different lowercase letters (*p* < 0.05).

Peanuts were wet-crushed, enzymatically digested, and then centrifuged to obtain OBEs and an aqueous and solid phase. Figure 2 illustrates the effects of the roasting temperature on the distribution of oil and proteins in each phase. The extraction rate of peanut oil, obtained through AEE, exhibited a trend of gradual increase followed by a rapid decrease as the roasting temperature increased. The highest peanut oil extraction rate reached 92.21% at 90 °C. The rate of residual oil in the aqueous and solid phases showed a trend of slight reduction followed by a sharp increase with increasing roasting temperature (Figure 2A). At 90 °C, the residual oil rates in the aqueous and solid phases were lower than other roasting temperatures, at 3.32% and 4.32%, respectively. This indicates that appropriate pre-roasting of peanuts is advantageous for extracting peanut oil.

**Figure 3:**
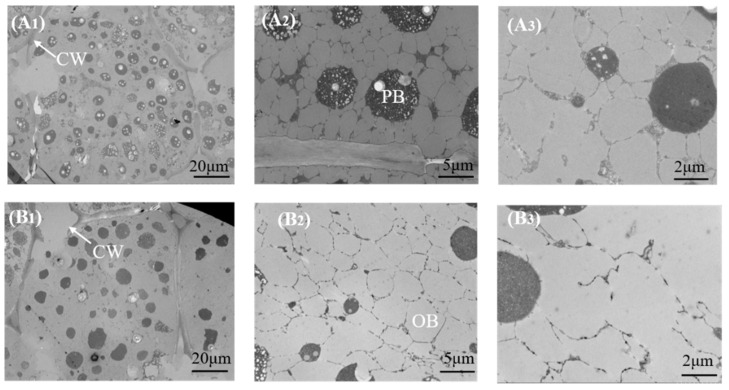
Observation of peanut microstructure at different roasting temperatures. (**A_1_**–**A_3_**), peanut cell structures at 50 °C; (**B_1_**–**B_3_**), peanut cell structures at 90 °C; OB, oil body; PB, protein body; CW, cell wall.

The protein extraction rate exhibited a pattern of gradual increase followed by a rapid decrease as the roasting temperature increased, reaching its peak at 90 °C, with a rate of 77.02% (Figure 2B). The protein content in the solid phase exhibited a minor decrease followed by a rapid increase as the roasting temperature increased, reaching a maximum of 55.56% at 150 °C, consistent with the findings of Li et al. [8]. Therefore, the optimal extraction of peanut oil and protein was achieved at a roasting temperature of 90 °C. As shown in Figure 3, compared to the peanut cell microstructure (A_1_) at 50 °C, the degree of cell wall rupture in peanuts was more pronounced in the cell wall structure at 90 °C (B_1_). These peanuts were more thoroughly crushed during wet processing, resulting in a greater degree disruption of their cellular structure. The enlarged contact area between peanuts and the wall-breaking enzyme accelerated the enzyme digestion process, promoting the release of oils and proteins and ultimately enhancing the extraction rate of peanut oil and protein [18,29]. Compared to 50 °C OB, the diameter of the 90 °C OB was significantly larger, potentially resulting from heat treatment causing some protein denaturation and the subsequent release of original bound oil and OB aggregation fusion (Figure 3A_3_,B_3_). At roasting temperatures > 90 °C, a significant degree of protein denaturation occurred, leading to reduced protein solubility. Consequently, a portion of the oil became trapped by insoluble proteins in the solid phase, resulting in a reduction in the rate of peanut oil and protein extraction.

### 3.2. Effect of Roasting Temperatures on the Composition of OBE

**Figure 4:**
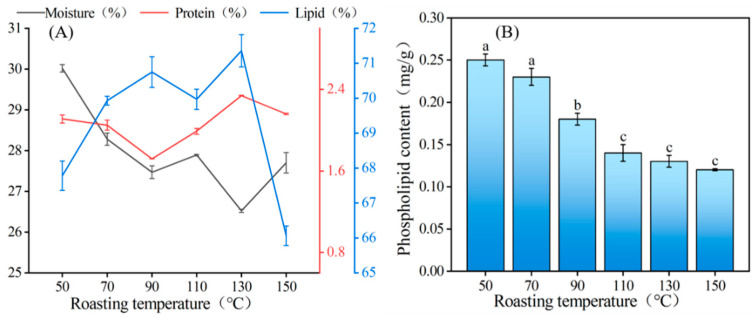
Changes in major components of peanut OBE under different roasting temperatures. (**A**) water, protein, and lipid; (**B**) phospholipid. A significant difference between samples is indicated by different lowercase letters (*p* < 0.05).

Understanding how extraction conditions affect OBE composition is crucial [30,31], emphasizing the significance of exploring the changes in major components of peanut OBEs extracted via AEE at different roasting temperatures. The main constituents of the OBEs derived from peanuts roasted at different temperatures using AEE were lipids, proteins, water, and phospholipids. However, the relative contents of the components varied markedly, affecting the stability of the OBEs. Figure 4A shows the results. The OBE exhibited its highest water content at 30.02% when roasted at 50 °C. The moisture content in the OBE decreased sharply between 50 °C and 90 °C. This trend may be attributed to the water-binding capacity of proteins. The different moisture contents of OBEs are thought to be from their different protein contents [32,33].

The protein content of the OBEs showed a fluctuating trend throughout the experimental temperature range. The protein content of the OBEs varied in the range of 1.72–2.34%, which is very small and consistent with the description of Gao et al. [12]. The protein content of the OBEs was relatively lowest when 90 °C was reached. The mechanism of the effect of roasting temperature on the protein content of OBEs is more complex. We speculate that the roasting temperature induces protein denaturation, leading to changes in protein structure, but the effect of changes in protein structure on the protein content of OBEs needs to be studied in depth.

The lipid composition of OBEs primarily comprises neutral lipids and a small fraction of phospholipids (Figure 4A). The relative lipid content exhibited a gradual increase with an escalation in roasting temperature, possibly owing to enhanced cell fragmentation and augmented release rate of peanut OBs. At a roasting temperature of 150 °C, the lipid relative content of the OBE decreased sharply, potentially attributed to an increased entrapment of lipids by insoluble proteins into the solid phase. As shown in Figure 4B, the phospholipid content showed a decreasing trend with increasing roasting temperature, possibly stemming from the increased destruction of peanut tissues and the release of various endogenous enzymes, including esterases and phospholipases. This enzymatic activity is known to degrade phospholipids, ultimately resulting in a decrease in the phospholipid content [34].

### 3.3. Effect of Roasting Temperature on the Stability of Peanut OBEs

**Figure 5:**
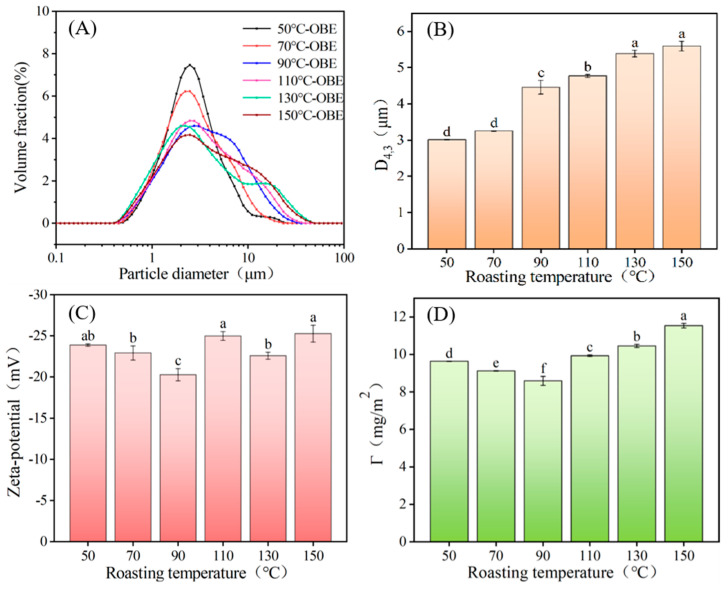
Effect of roasting temperature on the stability of OBEs. (**A**) Particle size distribution; (**B**) volume average particle size; (**C**) zeta potential; and (**D**) surface protein concentration. A significant difference between samples is indicated by different lowercase letters (*p* < 0.05).

The protein and phospholipid contents of peanut OBEs, prepared using peanuts at different roasting temperatures, exhibited significant variations, potentially affecting the stability of the OBEs. Therefore, the effect of different roasting temperatures on the stability of the OBEs was analyzed by comparing the D_4,3_, surface protein concentrations, and zeta potentials of the OBEs at different roasting temperatures. Figure 5 shows the results. The higher the absolute value of the zeta potential of the emulsion, the higher the electrostatic repulsion between the droplets. This also enhanced the stability of the emulsion, making it more resistant to breaking. Conversely, the lower the absolute value of the zeta potential—owing to the lack of electrostatic repulsion between droplets—the greater the aggregation between droplets, larger emulsion particle size, and increased susceptibility to emulsion breakdown [22]. Therefore, particle size and zeta potential are common indicators for assessing emulsion stability. OBs were stable and independent in peanut cotyledon cells; however, after roasting and processing, some of the OB structures ruptured and aggregated, resulting in an increased particle size. As can be seen from Figure 5A, the 50 °C OBE exhibited a single-peak distribution in particle size, with D_4,3_ of 3.014 μm, significantly larger than the reported diameter of the peanut oil body of approximately 1.95 μm according to Tzen et al. [35]. This suggests that roasting is beneficial for the aggregation and fusion of OBs. As the roasting temperature increased, the OB particle size exhibited a gradual bimodal distribution and shifted to higher values. As the roasting temperature increased, the D_4,3_ of the OBEs gradually increased. Furthermore, the D_4,3_ of OBEs at 70 °C, 90 °C, 110 °C, 130 °C, and 150 °C were 3.253, 4.461, 4.773, 5.390, and 5.595 μm, respectively (Figure 5B). This highlights the significant effect of roasting temperature on the OBE particle size. Figure 5C illustrates the zeta potentials of the OBEs extracted at different roasting temperatures. The zeta potential for OBEs roasted at 50 °C, 70 °C, 90 °C, 110 °C, 130 °C, and 150 °C was measured at −23.88, −22.92, −20.28, −24.99, −22.59, and −25.27 mV, respectively. The absolute value of the 90 °C OBE zeta potential was significantly lower than the absolute values of the potentials for the remaining roasting temperatures [33].

OBE is a natural oil-in-water emulsion, and the surface protein concentration serves as one of the critical factors affecting emulsion stability. A greater surface protein concentration indicates a higher degree of protein coverage on the surface film of the oil film, leading to a reduced interfacial tension between the two phases and consequently enhancing the stability of the emulsion [36]. Figure 5D shows that with the increased roasting temperature, the surface protein concentration of the OBs showed a trend of initially decreasing and subsequently increasing with the elevation of roasting temperature. The surface protein concentration for OBEs roasted at 50 °C, 70 °C, 90 °C, 110 °C, 130 °C, and 150 °C were 9.63, 9.12, 8.59, 9.93, 10.45, and 11.53 mg/m^2^, respectively. These values exhibited significant disparities among OBEs subjected to different roasting temperatures, with the 90 °C OBE showing the lowest surface protein concentration. Tcholakova et al. [37] found that when the surface protein concentration of oil droplets ranged from 1 to 2 mg/m^2^, it resulted in the formation of an emulsion stabilized by a single protein film. However, the surface protein concentration of OBE at different roasting temperatures was significantly higher than the minimum surface protein concentration required for oil droplets. This indicates the potential formation of multilayered protein films during AEE. Considering the particle size and zeta potential results, it is clear that the stability of the 90 °C OBE was inferior to that of the OBEs obtained from the other roasting temperatures. The low efficiency of AEE in demulsifying emulsions remains one of the significant obstacles to its industrial adoption. The lower the stability of OBEs, the lower the challenge of extracting peanut oil via subsequent demulsification with AEE. This study on the stability of peanut OBEs provides a theoretical basis for the selection of pre-roasting temperature for the simultaneous extraction of peanut oil and protein with AEE.

### 3.4. Microstructure of OBEs

**Figure 6:**
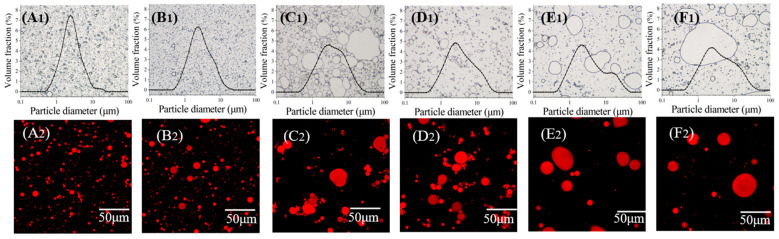
Microscopic observation of OBEs under different roasting temperatures. (**A**–**F**) OBEs under 50, 70, 90, 110, 130, and 150 °C temperatures, respectively.

Figure 6 presents the microscopic observations of the OBEs at various roasting temperatures. The particle size distribution of the OBE at 50 °C appears unimodal, with a predominant concentration in the 1–10 μm range (Figure 6A_1_). All the oil droplets maintained a circular uniform size. As the roasting temperature increased, a higher proportion of oil volume was distributed in particles ranging from 10 to 100 µm; at the same time, a gradual decrease in oil volume distributed in smaller particles (1–10 µm) was observed. Most of the 70 °C OB retained its rounded shape and uniform size (Figure 6B_1_). This observation is consistent with that in Figure 5B, indicating no significant difference in the D_4,3_ between the 50 °C and 70 °C OBEs. Furthermore, the 90 °C OB demonstrated coalescence destabilization, displaying an irregular shape, although a few oil droplets remained unchanged in size (Figure 6C_1_). Figure 6D_1_ shows that most of the 110 °C OB showed aggregation. Figure 6E_1_ depicts the micrograph of the 130 °C OBE, with a bimodal distribution of particle sizes and two peaks at 1–10 μm and 10–100 μm, respectively, indicating that some oil droplets coalesced and increased in size. Figure 6F_1_ shows the 150 °C OBE micrograph characterized by a bimodal distribution. CLSM was used to observe the microstructure of the OBE. The central area of the OBs was stained with Nile red, exhibiting red fluorescence, indicating its richness in oil content. Similar to the results observed under a microscope, the OB at 50 °C exhibited a spherical shape of uniform size. However, as the roasting temperature increased, the oil droplets aggregated.

### 3.5. Effect of Different Roasting Temperatures on Peanut Oil Quality

#### 3.5.1. Analysis of Physicochemical Indicators and Tocopherol Content

Table 1 shows the physicochemical properties and tocopherol content of peanut oil extracted through pressing, leaching, commercial availability, and various roasting temperatures using AEE. The acid value and peroxide value of peanut oil extracted through AEE at varying roasting temperatures were lower than those of pressed oil. Subsequently, accelerated oxidation experiments were conducted using peanut oil extracted using different extraction methods. Under high-temperature conditions, water, and oxygen, the triglycerides in the oils underwent hydrolysis, isomerization, and polymerization. Oxygen was continuously introduced into the oils at these elevated temperatures to detect the inflection point on the oxidation curve, which is the oxidation induction time. It is employed to assess the oxidative stability of oils and is frequently utilized to predict the shelf life of oils [38]. The oxidative stability of peanut oil extracted using AEE followed a pattern of initially decreasing slightly and then increasing with rising roasting temperature. The longer the induction time, the better the oxidative stability. Oils extracted from roasted sunflowers, rapeseed, and walnut kernels also exhibit increased oxidative stress [39,40]. The oxidative stability of peanut oil extracted through 70 °C AEE showed a slight reduction compared to those extracted using 50 °C. This decrease may be due to the higher roasting temperature, potentially degrading the natural antioxidant components within the peanut oil, while peanuts under this roasting temperature condition did not show an obvious Maillard reaction, and as the number of Maillard reaction products with antioxidant activity was low, which could not counteract the destroyed natural antioxidant components in the peanut oil, the antioxidant activity decreased. As the roasting temperature increased, the Maillard reaction accelerated, leading to an augmented production of Maillard reaction products with antioxidant activity. This increase was sufficient to neutralize the degradation of natural antioxidant components in the peanut oil, consequently enhancing the overall antioxidant activity. No significant differences were observed in the acid value, peroxide value, and oxidative stability of peanut oil extracted through 50 °C and 90 °C AEE, indicating that the quality of peanut oil extracted through 50 °C and 90 °C AEE was comparable.

Tocopherol isomers found in vegetable oils include *δ*-tocopherol, *β*-tocopherol, *γ*-tocopherol, and *α*-tocopherol, with *α*-tocopherol and *γ*-tocopherol being the major tocopherols in peanut oil [41]. The total tocopherol content in peanut oil extracted using AEE at 50, 70, and 90 °C was slightly higher than that of pressed, leached, and commercially available oils. This may be because tocopherols existed in the peanut OBs and the part of the OBs released by the wall-breaking enzyme enzymatically dismantling the cell wall was not destroyed, which was able to reduce the loss of tocopherols [25]. As the roasting temperature increased, tocopherol decomposition occurred, leading to a decrease in content [6]. This is consistent with the findings in Table 1, where the oxidative stability of water enzyme-extracted peanut oil initially decreased with increasing roasting temperature. The total tocopherol content in AEE peanut oil extracted from peanuts roasted at 50 °C and 90 °C for 30 min was 25.24 mg/100 g and 24.56 mg/100 g, respectively, which indicates that high temperature led to tocopherol loss. However, the loss was relatively low, accounting for only 2.7%.

#### 3.5.2. Analysis of Fatty Acid Composition

To investigate the effect of various roasting temperatures on the fatty acid composition of peanut oil extracted via AEE, the fatty acid composition of peanut oil obtained from treating peanuts at different roasting temperatures was analyzed and compared with those of pressed, leached, and commercially available oils. Table 2 presents the results. The fatty acid composition of peanut oil extracted through AEE at various roasting temperatures differed from that of pressed and commercially available oils. Arachidonic acid was detected in pressed oil but not in peanut oil extracted through other treatment methods. Trans-linoleic acid was found in commercially available oils but not in peanut oil extracted via other extraction processes. Overall, 12 fatty acids were identified in peanut oil extracted using AEE. These included three monounsaturated fatty acids, two polyunsaturated fatty acids, and seven saturated fatty acids. Among the saturated fatty acids, palmitic and stearic acids were the most abundant. Among the monounsaturated fatty acids, oleic acid was the most prevalent, accounting for approximately 37.37–39.02% of the total fatty acid content. This particular acid reduces LDL cholesterol levels in the blood and lowers blood pressure. Additionally, linoleic acid was the most abundant polyunsaturated fatty acid, accounting for 37.28–41.05% of the total fatty acid content. The oleic acid content in the AEE-extracted peanut oil decreased slightly with increasing roasting temperature. This indicates that roasting had a weak effect on the oleic acid content of peanut oil. The fatty acid composition of peanut oil extracted through AEE at different roasting temperatures did not exhibit any significant variation, consistent with previous findings that suggest roasting has no significant effect on the fatty acid composition of the oil [40,42,43].

The consumption of trans fatty acids is positively associated with the development of cancers, such as breast and colon cancers and cardiovascular disease [44]. While trans-oleic and trans-linoleic acids were found in commercial oils, only trans-linoleic acid was detected in the AEE-extracted peanut oils, and its content was significantly lower than that in the commercial oils. The unsaturated fatty acid content of peanut oil extracted via AEE ranged from approximately 77.09 to 79.21%, higher than that of pressed and leached oils but slightly lower than that of commercial oils. The ratio of oleic acid to linoleic acid (O/L) is commonly used as a stability index to evaluate the quality of peanuts and their products. From a contemporary nutritional perspective, a high O/L value represents a product with a high nutritional value [20,45]. No significant differences were observed in the saturated and unsaturated fatty acid contents and O/L value between peanut oils extracted using the 50 °C AEE and those extracted via the 90 °C AEE approach.

### 3.6. Effect of Different Roasting Temperatures on Protein Quality

#### 3.6.1. Effect of Different Roasting Temperatures on Polypeptide Composition

**Figure 7:**
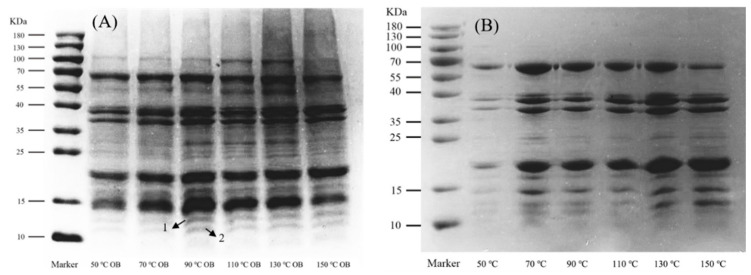
Protein composition at different roasting temperatures. (**A**) The OBIP composition; (**B**) the composition of peanut protein using AEE.

During the AEE process of peanut oil, some exogenous proteins are adsorbed on the surface of the OB. The composition and content of these proteins affect their stability during storage and processing. To gain insight into these components, the composition of OBIPs and peanut proteins extracted via AEE at different roasting temperatures was analyzed using SDS-PAGE. Figure 7 shows the results. There was almost no difference in OBIP composition extracted at different roasting temperatures and relative contents (Figure 7A). This indicates that the roasting temperature exerted a minimal effect on the composition and relative content of OBIPs. The content of bands 1 and 2 in the OBIPs extracted through AEE at 50 °C and 70 °C was slightly lower than that of OBIPs extracted via AEE at other roasting temperatures. This may be due to the increase in roasting temperature, leading to some protein denaturation and the exposure of internal hydrophobic groups, which increased the binding to the surface of the OBs. The composition of peanut protein extracted via AEE at different roasting temperatures showed minimal variations, indicating that roasting temperature had a negligible effect on peanut protein composition (Figure 7B).

#### 3.6.2. Effect of Different Roasting Temperatures on Amino Acid Composition

The amino acid compositions of peanut proteins extracted at different roasting temperatures were determined. Table 3 shows the results. Amino acids play a pivotal role as primary contributors to the Maillard reaction and serve as fundamental precursors to flavor compounds, significantly contributing to the quality and value of peanuts. There was no difference in the amino acid composition of peanut proteins extracted at varying roasting temperatures, and these proteins were abundant in essential amino acids. Peanut protein extracted at 50 °C exhibited elevated levels of glutamic acid, aspartic acid, and arginine (21.16%, 12.64%, and 9.32%, respectively) and lower levels of cysteine and proline (0.67% and 0.31%, respectively). Among the 17 amino acids detected, lysine and arginine showed the most substantial decrease. After roasting at 90 °C for 30 min, the lysine content decreased from 3.50% to 3.25% at 50 °C roasting, resulting in a loss rate of >7.14%. Similarly, the arginine content decreased from 9.32% to 9.21% at 50 °C roasting, reflecting a loss rate of approximately 1.2%. In contrast, when roasted at 150 °C, the lysine content dropped from 3.50% to 2.76% at 50 °C roasting, leading to a loss rate of ≥21%. The arginine content, when roasted at 150 °C, decreased from 9.32% to 8.83% when roasted at 50 °C, indicating a loss rate of approximately 5.3%. Lysine and arginine are hypothesized to potentially serve as precursor substrates in the Maillard reaction [40]. Hydrophobic amino acids, including alanine, valine, methionine, isoleucine, leucine, phenylalanine, and proline, are found within proteins, providing hydrophobic interactions to maintain their tertiary structure. The essential amino acid content ranged from 29.60 to 30.60%, while the hydrophobic amino acid content ranged from 29.13 to 31.92%. Moreover, there was no significant difference between the essential and hydrophobic amino acid contents in peanut proteins extracted at different roasting temperatures (Table 3).

## 4. Conclusions

According to the findings, the highest yields of peanut oil and protein, at 92.21% and 77.02%, respectively, were obtained from peanuts after roasting treatment at 90 °C. While the roasting temperature did not affect the composition of peanut OBEs, variations were observed in the relative content of each constituent. By comparing the particle size, zeta potential, and surface protein concentration of peanut OBEs extracted from varying roasting temperatures and observing their microstructures, OBEs were found to exhibit the lowest stability at 90 °C. These characteristics make them more suitable for subsequent emulsion breaking for peanut oil extraction. In conclusion, this study can provide a better understanding of the mechanism by which roasting temperature affects the simultaneous extraction of peanut oil and protein using AEE.

## Figures and Tables

**Table 1:** Physicochemical indexes and tocopherol content of peanut oil extracted by different treatments.

Constituent	Pressed Oil	Leaching Oil	Commercial Oil	AEE					
				50 °C	70 °C	90 °C	110 °C	130 °C	150 °C
Acid value/(mg KOH/g)	1.33 ± 0.01 ^a^	0.12 ± 0.007 ^e^	0.58 ± 0.02 ^b^	0.18 ± 0.005 ^d^	0.22 ± 0.03 ^d^	0.22 ± 0.02 ^d^	0.2 ± 0.03 ^d^	0.2 ± 0.02 ^d^	0.35 ± 0.02 ^c^
Peroxide value/(g/100 g)	0.19 ± 0.002 ^a^	0.03 ± 0.002 ^d^	0.1 ± 0.005 ^b^	0.12 ± 0.002 ^b^	0.07 ± 0.002 ^c^	0.09 ± 0.002 ^b^	0.1 ± 0.007 ^b^	0.09 ± 0.002 ^b^	0.06 ± 0.002 ^c^
Oxidation stability/(h)	2.44 ± 0.05 ^c^	4.46 ± 0.02 ^a^	3.25 ± 0.08 ^b^	0.88 ± 0.04 ^g^	0.63 ± 0.01 ^h^	0.86 ± 0.06 ^g^	2.2 ± 0.03 ^d^	1.37 ± 0.01 ^f^	1.8 ± 0.06 ^e^
Tocopherols (mg/100 g)									
α	10.51 ± 0.23 ^c^	10.88 ± 0.48 ^bc^	10.74 ± 0.03 ^bc^	11.49 ± 0.13 ^a^	11.17 ± 0.04 ^ab^	10.97 ± 0.04 ^bc^	10.78 ± 0.08 ^bc^	10.59 ± 0.03 ^c^	9.86 ± 0.21 ^d^
γ	12.46 ± 0.16 ^d^	12.59 ± 0.04 ^cd^	12.46 ± 0.30 ^d^	13.22 ± 0.14 ^a^	12.96 ± 0.03 ^ab^	13.01 ± 0.10 ^ab^	12.96 ± 0.16 ^ab^	12.86 ± 0.03 ^bc^	12.96 ± 0.11 ^ab^
δ	0.98 ± 0.06 ^a^	1.02 ± 0.07 ^a^	1.00 ± 0.16 ^a^	1.03 ± 0.03 ^a^	1.00 ± 0.04 ^a^	1.06 ± 0.03 ^a^	0.98 ± 0.13 ^a^	0.87 ± 0.01 ^a^	1.00 ± 0.01 ^a^
Total contents	23.48 ± 0.41 ^cd^	24.00 ± 0.56 ^bcd^	23.71 ± 0.41 ^cd^	25.24 ± 0.29 ^a^	24.69 ± 0.09 ^ab^	24.56 ± 0.16 ^ab^	24.25 ± 0.31 ^bc^	23.90 ± 0.06 ^bcd^	23.36 ± 0.33 ^d^

Note: A significant difference between samples is indicated by different lowercase letters (*p* < 0.05).

**Table 2:** Fatty acid composition of peanut oil extracted at different roasting temperatures.

Fatty Acid	Pressed Oil	Leaching Oil	Commercial Oil	AEE					
				50 °C	70 °C	90 °C	110 °C	130 °C	150 °C
C14:0	0.03 ± 0.00 ^c^	0.19 ± 0.00 ^a^	0.04 ± 0.00 ^b^	0.03 ± 0.00 ^c^	0.03 ± 0.00 ^c^	0.03 ± 0.00 ^c^	0.03 ± 0.00 ^c^	0.03 ± 0.00 ^c^	0.03 ± 0.00 ^c^
C16:0	12.56 ± 0.20 ^c^	12.36 ± 0.02 ^c^	9.89 ± 0.02 ^e^	12.78 ± 0.01 ^b^	13.29 ± 0.01 ^a^	13.08 ± 0.01 ^a^	12.41 ± 0.01 ^c^	12.03 ± 0.01 ^d^	12.10 ± 0.00 ^d^
C16:1	0.09 ± 0.00 ^a^	0.04 ± 0.00 ^c^	0.06 ± 0.00 ^b^	0.04 ± 0.00 ^c^	0.04 ± 0.00 ^c^	0.04 ± 0.00 ^c^	0.05 ± 0.00 ^c^	0.04 ± 0.00 ^c^	0.04 ± 0.00 ^c^
C17:0	0.07 ± 0.01 ^a^	0.04 ± 0.00 ^b^	0.05 ± 0.00 ^b^	0.05 ± 0.00 ^b^	0.05 ± 0.00 ^b^	0.05 ± 0.00 ^b^	0.05 ± 0.00 ^b^	0.05 ± 0.00 ^b^	0.05 ± 0.00 ^b^
C18:0	4.13 ± 0.04 ^a^	3.93 ± 0.01 ^b^	3.58 ± 0.00 ^d^	4.06 ± 0.02 ^a^	4.09 ± 0.00 ^a^	4.13 ± 0.00 ^a^	3.87 ± 0.00 ^b^	3.74 ± 0.00 ^c^	3.81 ± 0.01 ^b^
CT18:1	-	-	0.04 ± 0.00 ^a^	-	-	-	-	-	-
C18:1	50.60 ± 0.00 ^a^	38.93 ± 0.03 ^d^	48.88 ± 0.00 ^b^	39.00 ± 0.07 ^c^	39.02 ± 0.03 ^c^	38.31 ± 0.02 ^e^	37.92 ± 0.03 ^f^	37.37 ± 0.03 ^h^	37.60 ± 0.01 ^g^
CT18:2	0.02 ± 0.00 ^c^	0.02 ± 0.00 ^c^	0.12 ± 0.00 ^a^	0.03 ± 0.00 ^b^	0.03 ± 0.00 ^b^	0.03 ± 0.00 ^b^	0.03 ± 0.00 ^b^	0.03 ± 0.00 ^b^	0.03 ± 0.00 ^b^
C18:2	25.21 ± 0.27 ^d^	39.20 ± 0.05 ^a^	31.71 ± 0.02 ^c^	37.76 ± 0.05 ^b^	37.28 ± 0.03 ^b^	38.31 ± 0.03 ^b^	39.89 ± 0.01 ^a^	41.05 ± 0.02 ^a^	40.79 ± 0.03 ^a^
C20:0	1.62 ± 0.02 ^a^	1.44 ± 0.05 ^c^	1.26 ± 0.01 ^d^	1.62 ± 0.00 ^a^	1.62 ± 0.01 ^a^	1.61 ± 0.00 ^a^	1.54 ± 0.00 ^b^	1.49 ± 0.02 ^c^	1.48 ± 0.01 ^c^
C20:1	0.92 ± 0.00 ^a^	0.70 ± 0.01 ^b^	0.91 ± 0.02 ^a^	0.78 ± 0.00 ^b^	0.76 ± 0.00 ^b^	0.75 ± 0.01 ^b^	0.74 ± 0.00 ^b^	0.75 ± 0.01 ^b^	0.72 ± 0.01 ^b^
C22:0	3.22 ± 0.03 ^a^	2.11 ± 0.03 ^c^	2.40 ± 0.02 ^b^	2.42 ± 0.00 ^b^	2.41 ± 0.00 ^b^	2.39 ± 0.01 ^b^	2.29 ± 0.00 ^b^	2.25 ± 0.01 ^bc^	2.22 ± 0.00 ^c^
C20:3	0.08 ± 0.00 ^a^	-	-	-	-	-	-	-	-
C24:0	1.44 ± 0.00 ^a^	1.02 ± 0.02 ^d^	1.06 ± 0.01 ^cd^	1.42 ± 0.09 ^a^	1.39 ± 0.07 ^ab^	1.30 ± 0.03 ^b^	1.18 ± 0.02 ^c^	1.16 ± 0.02 ^c^	1.13 ± 0.01 ^c^
MUFA	51.61 ± 0.01 ^a^	39.67 ± 0.02 ^c^	49.85 ± 0.02 ^b^	39.82 ± 0.07 ^c^	39.82 ± 0.03 ^c^	39.09 ± 0.00 ^d^	38.71 ± 0.02 ^e^	38.16 ± 0.02 ^e^	38.36 ± 0.00 ^e^
PUFA	25.29 ± 0.27 ^f^	39.20 ± 0.05 ^c^	31.71 ± 0.02 ^e^	37.76 ± 0.05 ^d^	37.28 ± 0.03 ^d^	38.31 ± 0.03 ^d^	39.89 ± 0.01 ^c^	41.05 ± 0.02 ^a^	40.79 ± 0.03 ^b^
UFA	76.90 ± 0.28 ^e^	78.87 ± 0.07 ^c^	81.56 ± 0.00 ^a^	77.58 ± 0.11 ^d^	77.09 ± 0.06 ^e^	77.40 ± 0.03 ^d^	78.60 ± 0.04 ^c^	79.21 ± 0.04 ^b^	79.15 ± 0.02 ^b^
SFA	23.08 ± 0.28 ^a^	21.10 ± 0.07 ^c^	18.28 ± 0.00 ^d^	22.39 ± 0.11 ^b^	22.88 ± 0.06 ^a^	22.58 ± 0.03 ^b^	21.38 ± 0.04 ^c^	20.76 ± 0.04 ^c^	20.83 ± 0.02 ^c^
O/L	2.01 ± 0.02 ^a^	0.99 ± 0.00 ^c^	1.54 ± 0.00 ^b^	1.03 ± 0.00 ^c^	1.05 ± 0.00 ^c^	1.00 ± 0.00 ^c^	0.95 ± 0.00 ^d^	0.91 ± 0.00 ^d^	0.92 ± 0.00 ^d^

Note: MUFA, PUFA, UFA, and SFA represent monounsaturated fatty acids, polyunsaturated fatty acids, unsaturated fatty acids, and saturated fatty acids, respectively. A significant difference between samples is indicated by different lowercase letters (*p* < 0.05).

**Table 3:** Amino acid composition of peanut proteins extracted at different roasting temperatures.

Amino Acid	Roast Temperature (°C)					
	50	70	90	110	130	150
Asp	12.64 ± 0.09 ^ab^	12.87 ± 0.04 ^a^	12.75 ± 0.34 ^a^	12.42 ± 0.30 ^ab^	12.40 ± 0.46 ^ab^	12.08 ± 0.01 ^b^
Thr	2.57 ± 0.44 ^b^	3.26 ± 0.01 ^a^	3.03 ± 0.15 ^ab^	3.02 ± 0.04 ^ab^	2.92 ± 0.05 ^ab^	2.97 ± 0.04 ^ab^
Ser	6.36 ± 0.38 ^c^	6.91 ± 0.03 ^ab^	7.08 ± 0.10 ^a^	6.66 ± 0.01 ^abc^	6.55 ± 0.04 ^bc^	6.56 ± 0.08 ^bc^
Glu	21.16 ± 0.26 ^a^	20.92 ± 0.12 ^a^	20.92 ± 0.21 ^a^	20.74 ± 0.34 ^a^	20.79 ± 0.23 ^a^	21.24 ± 0.04 ^a^
Gly	8.34 ± 0.07 ^ab^	8.09 ± 0.07 ^ab^	8.00 ± 0.13 ^b^	8.12 ± 0.23 ^ab^	7.94 ± 0.38 ^b^	8.48 ± 0.02 ^a^
Ala	5.67 ± 0.20 ^a^	5.52 ± 0.08 ^ab^	5.60 ± 0.15 ^ab^	5.51 ± 0.29 ^ab^	5.48 ± 0.23 ^ab^	5.21 ± 0.01 ^b^
Cys	0.67 ± 0.31 ^a^	0.81 ± 0.08 ^a^	0.78 ± 0.02 ^a^	0.68 ± 0.02 ^a^	0.68 ± 0.03 ^a^	0.90 ± 0.00 ^a^
Val	5.02 ± 0.37 ^ab^	5.06 ± 0.04 ^a^	5.11 ± 0.01 ^a^	4.95 ± 0.15 ^ab^	4.88 ± 0.15 ^ab^	4.53 ± 0.04 ^b^
Met	2.24 ± 0.07 ^ab^	2.22 ± 0.11 ^b^	2.05 ± 0.08 ^b^	2.49 ± 0.65 ^ab^	2.67 ± 0.87 ^ab^	3.25 ± 0.10 ^a^
Ile	3.97 ± 0.04 ^a^	3.91 ± 0.02 ^a^	3.93 ± 0.03 ^a^	3.94 ± 0.14 ^a^	3.99 ± 0.13 ^a^	3.83 ± 0.02 ^a^
Leu	7.69 ± 0.01 ^a^	7.49 ± 0.03 ^a^	7.59 ± 0.00 ^a^	7.75 ± 0.26 ^a^	7.93 ± 0.45 ^a^	7.75 ± 0.04 ^a^
Tyr	3.20 ± 0.04 ^b^	3.15 ± 0.02 ^b^	3.20 ± 0.03 ^b^	3.47 ± 0.18 ^a^	3.46 ± 0.13 ^a^	3.54 ± 0.01 ^a^
Phe	4.72 ± 0.04 ^ab^	4.61 ± 0.03 ^b^	4.66 ± 0.00 ^b^	5.12 ± 0.49 ^ab^	5.32 ± 0.44 ^a^	5.01 ± 0.06 ^ab^
His	2.59 ± 0.06 ^a^	2.50 ± 0.09 ^a^	2.52 ± 0.01 ^a^	2.57 ± 0.15 ^a^	2.63 ± 0.12 ^a^	2.65 ± 0.00 ^a^
Lys	3.50 ± 0.05 ^a^	3.38 ± 0.02 ^a^	3.25 ± 0.01 ^b^	3.09 ± 0.10 ^c^	2.89 ± 0.09 ^d^	2.76 ± 0.00 ^e^
Arg	9.32 ± 0.11 ^a^	8.97 ± 0.13 ^a^	9.21 ± 0.07 ^a^	9.02 ± 0.43 ^a^	9.02 ± 0.45 ^a^	8.83 ± 0.03 ^a^
Pro	0.31 ± 0.13 ^a^	0.33 ± 0.01 ^a^	0.35 ± 0.19 ^a^	0.45 ± 0.01 ^a^	0.45 ± 0.02 ^a^	0.42 ± 0.00 ^a^
EAA	29.72 ± 0.13 ^a^	29.93 ± 0.13 ^a^	29.60 ± 0.26 ^a^	30.35 ± 1.26 ^a^	30.60 ± 1.59 ^a^	30.09 ± 0.11 ^a^
HAA	29.63 ± 0.07 ^a^	29.13 ± 0.07 ^a^	29.28 ± 0.45 ^a^	30.22 ± 1.11 ^a^	31.92 ± 3.18 ^a^	29.98 ± 0.17 ^a^

Note: EAA and HAA represent essential amino acids and hydrophobic amino acids, respectively. A significant difference between samples is indicated by different lowercase letters (*p* < 0.05).

## Data Availability

Relevant information and techniques have been provided in this study. The corresponding author should be contacted for any further questions.

## References

[1] https://apps.fas.usda.gov/psdonline/app/index.html#/app/advQuery.

[2] Niu R.H., Chen F.S., Liu C., Duan X.J. (2021). Composition and Rheological Properties of Peanut Oil Bodies from Aqueous Enzymatic Extraction. J. Oleo Sci..

[3] Mahfoud F., Assaf J.C., Elias R., Debs E., Louka N. (2023). Defatting and Defatted Peanuts: A Critical Review on Methods of Oil Extraction and Consideration of Solid Matrix as a By-Product or Intended Target. Processes.

[4] Liu C., Chen F.S., Xia Y.M., Liu B.Y. (2022). Physicochemical and rheological properties of peanut oil body following alkaline pH treatment. LWT-Food Sci. Technol..

[5] Yusoff M.M., Gordon M.H., Ezeh O., Niranjan K. (2016). Aqueous enzymatic extraction of Moringa oleifera oil. Food Chem..

[6] Shrestha K., De Meulenaer B. (2014). Effect of Seed Roasting on Canolol, Tocopherol, and Phospholipid Contents, Maillard Type Reactions, and Oxidative Stability of Mustard and Rapeseed Oils. J. Agric. Food Chem..

[7] Wijesundera C., Ceccato C., Fagan P., Shen Z.P. (2008). Seed roasting improves the oxidative stability of canola (B-napus) and mustard (B-juncea) seed oils. Eur. J. Lipid Sci. Technol..

[8] Li P.F., Gasmalla M.A.A., Zhang W.B., Liu J.J., Bing R., Yang R.J. (2016). Effects of roasting temperatures and grinding type on the yields of oil and protein obtained by aqueous extraction processing. J. Food Eng..

[9] Huang A.H.C. (2018). Plant Lipid Droplets and Their Associated Proteins: Potential for Rapid Advances. Plant Physiol..

[10] Barbosa A.D., Siniossoglou S. (2017). Function of lipid droplet-organelle interactions in lipid homeostasis. Biochim. Biophys. Acta-Mol. Cell Res..

[11] Hu M., Du X.Q., Liu G.N., Tan Z., Zhang S., Qi B.K., Li Y. (2022). Investigation of structure-stability correlations of reconstructed oil bodies. LWT-Food Sci. Technol..

[12] Gao Y.H., Zheng Y.Z., Yao F., Chen F.S. (2022). Effects of pH and temperature on the stability of peanut oil bodies: New insights for embedding active ingredients. Colloids Surf. A-Physicochem. Eng. Asp..

[13] Zaaboul F., Raza H., Chen C., Liu Y.F. (2018). Characterization of Peanut Oil Bodies Integral Proteins, Lipids, and Their Associated Phytochemicals. J. Food Sci..

[14] Zaaboul F., Zhao Q.L., Xu Y.J., Liu Y.F. (2022). Soybean oil bodies: A review on composition, properties, food applications, and future research aspects. Food Hydrocoll..

[15] Zhao L.P., Chen Y.M., Chen Y.J., Kong X.Z., Hua Y.F. (2016). Effects of pH on protein components of extracted oil bodies from diverse plant seeds and endogenous protease-induced oleosin hydrolysis. Food Chem..

[16] Liu C., Chen F.S., Xia Y.M. (2022). Composition and structural characterization of peanut crude oil bodies extracted by aqueous enzymatic method. J. Food Compos. Anal..

[17] Zhou L.Z., Chen F.S., Hao L.H., Du Y., Liu C. (2019). Peanut Oil Body Composition and Stability. J. Food Sci..

[18] Liu C., Chen F.S., Niu R.H., Gao Y.H. (2020). Effects of Pretreatment on the Yield of Peanut Oil and Protein Extracted by Aqueous Enzymatic Extraction and the Characteristics of the Emulsion. J. Oleo Sci..

[19] Zhao Y.H., Chen F.S., Wang Y.Y. (2023). Demulsification of peanut emulsion by aqueous enzymatic extraction using a combination of oleic and citric acids. LWT-Food Sci. Technol..

[20] Xu D.X., Gao Q.R., Ma N.N., Hao J., Yuan Y.M., Zhang M., Cao Y.P., Ho C.T. (2021). Structures and physicochemical characterization of enzyme extracted oil bodies from rice bran. LWT-Food Sci. Technol..

[21] Chabrand R.M., Kim H.J., Zhang C., Glatz C.E., Jung S. (2008). Destabilization of the emulsion formed during aqueous extraction of soybean oil. J. Am. Oil Chem. Soc..

[22] Li P.F., Gasmalla M.A.A., Liu J.J., Zhang W.B., Yang R.J., Aboagarib E.A.A. (2016). Characterization and demusification of cream emulsion from aqueous extraction of peanut. J. Food Eng..

[23] Cao J.B., Song Y.T., Wu H., Qin L.H., Hu L.H., Hao R. (2013). Ultrastructural Studies on the Natural Leaf Senescence of Cinnamomum Camphora. Scanning.

[24] AOCS (2009). Official Methods and Recommended Practices ofthe American Oil Chemists’ Society.

[25] Gao Y.H., Zheng Y.Z., Yao F., Chen F.S. (2023). A Novel Strategy for the Demulsification of Peanut Oil Body by Caproic Acid. Foods.

[26] Ji J.M., Liu Y.L., Shi L.K., Wang N.N., Wang X.D. (2019). Effect of roasting treatment on the chemical composition of sesame oil. LWT-Food Sci. Technol..

[27] Zhou L.Z., Chen F.S., Liu K.L., Zhu T.W., Jiang L.Z. (2020). Combination of Alcalase 2.4 L and CaCl_2_ for aqueous extraction of peanut oil. J. Food Sci..

[28] Liu K.L., Liu Y., Chen F.S. (2018). Effect of gamma irradiation on the physicochemical properties and nutrient contents of peanut. Lwt-Food Sci. Technol..

[29] Vovk H., Karnpakdee K., Ludwig R., Nosenko T. (2023). Enzymatic Pretreatment of Plant Cells for Oil Extraction. Food Technol. Biotechnol..

[30] Yang J.C., Vardar U.S., Boom R.M., Bitter J.H., Nikiforidis C.V. (2023). Extraction of oleosome and protein mixtures from sunflower seeds. Food Hydrocoll..

[31] Nikiforidis C.V. (2019). Structure and functions of oleosomes (oil bodies). Adv. Colloid Interface Sci..

[32] Zhao L.P., Chen Y.M., Yan Z.H., Kong X.Z., Hua Y.F. (2016). Physicochemical and rheological properties and oxidative stability of oil bodies recovered from soybean aqueous extract at different pHs. Food Hydrocoll..

[33] Zhang S.B., Lu Q.Y. (2015). Characterizing the structural and surface properties of proteins isolated before and after enzymatic demulsification of the aqueous extract emulsion of peanut seeds. Food Hydrocoll..

[34] Barros M., Fleuri L.F., Macedo G.A. (2010). Seed Lipases: Sources, Applications And Properties—A Review. Braz. J. Chem. Eng..

[35] Tzen J.T.C., Cao Y., Laurent P., Ratnayake C., Huang A.H.C. (1993). Lipids, Proteins, and Structure of Seed Oil Bodies from Diverse Species. Plant Physiol..

[36] Castellani O., Belhomme C., David-Briand E., Guerin-Dubiard C., Anton M. (2006). Oil-in-water emulsion properties and interfacial characteristics of hen egg yolk phosvitin. Food Hydrocoll..

[37] Tcholakova S., Denkov N.D., Sidzhakova D., Ivanov I.B., Campbell B. (2003). Interrelation between drop size and protein adsorption at various emulsification conditions. Langmuir.

[38] Liu B.G., Du J.Q., Zeng J., Chen C.G., Niu S.Y. (2009). Characterization and antioxidant activity of dihydromyricetin-lecithin complex. Eur. Food Res. Technol..

[39] Gao P., Cao Y., Liu R.J., Jin Q.Z., Wang X.G. (2019). Phytochemical Content, Minor-Constituent Compositions, and Antioxidant Capacity of Screw-Pressed Walnut Oil Obtained from Roasted Kernels. Eur. J. Lipid Sci. Technol..

[40] Ji J.M., Zhang Y., Wang Y., Wang D., Jie H. (2023). Influence of seed-roasting degree on quality attributes of sunflower oil. J. Food Sci..

[41] Davis J.P., Dean L.L., Price K.M., Sanders T.H. (2010). Roast effects on the hydrophilic and lipophilic antioxidant capacities of peanut flours, blanched peanut seed and peanut skins. Food Chem..

[42] Li K.S., Ali M.A., Muhammad I.I., Othman N.H., Noor A.M. (2018). The Effect of Microwave Roasting Over the Thermooxidative Degradation of Perah Seed Oil During Heating. J. Oleo Sci..

[43] Fathi-Achachlouei B., Azadmard-Damirchi S., Zahedi Y., Shaddel R. (2019). Microwave pretreatment as a promising strategy for increment of nutraceutical content and extraction yield of oil from milk thistle seed. Ind. Crops Prod..

[44] Bykova O., Shevkunov O., Kostyunina O. (2023). Overview of SNPs Associated with Trans Fat Content in Cow’s Milk. Agriculture.

[45] Capellini M.C., Giacomini V., Cuevas M.S., Rodrigues C.E.C. (2018). Rice bran oil extraction using alcoholic solvents: Physicochemical characterization of oil and protein fraction functionality (vol 104, pg 133, 2017). Ind. Crops Prod..

